# The effects of incivility on learning burnout in Chinese nursing interns: mediating roles of career calling and self-efficacy

**DOI:** 10.3389/fpsyg.2025.1478849

**Published:** 2025-09-12

**Authors:** Ou Na, Qi Sanyang, Li Pengxin, Hu Jimei, Hu Minmin

**Affiliations:** ^1^Department of Oncology, Affiliated Nanhua Hospital, University of South China, Hengyang, Hunan, China; ^2^College of Nursing and Allied Health Sciences, St. Paul University Manila, Malate Manila, Philippines; ^3^Department of Psychology, The Fourth People’s Hospital of Shenzhen, Shenzhen, Guangdong, China; ^4^College of Nursing, University of South China, Hengyang, Hunan, China

**Keywords:** nursing interns, incivility, learning burnout, career calling, self-efficacy

## Abstract

**Introduction:**

Incivility in clinical nursing education (ICNE) is a pervasive issue globally, adversely affecting nursing interns’ psychological well-being and professional development. However, the mediating mechanisms linking ICNE to learning burnout remain underexplored, particularly in non-Western contexts. This study investigates the impact of incivility in clinical nursing education on learning burnout among Chinese nursing interns and examines the mediating roles of career calling and self-efficacy.

**Methods:**

Using a sample of 250 nursing interns from a tertiary hospital in Hunan Province, China, data were collected through validated questionnaires. Statistical analyses, including regression and structural equation modeling, were conducted to explore relationships among variables.

**Results:**

Incivility significantly increased learning burnout levels (*β* = 0.784, *p* < 0.001), explaining 78.6% of the variance. Career calling and self-efficacy were found to mediate this relationship, accounting for 27.3 and 20.6% of the indirect effects, respectively. The total indirect effect was 47.9%, while the direct effect was 52.1%.

**Conclusion:**

Incivility in clinical nursing education significantly contributes to learning burnout. Career calling and self-efficacy partially mediate this effect, suggesting that enhancing these factors may mitigate the negative impact of incivility. These findings highlight the importance of improving the educational environment and providing personalized support to nursing interns.

## 1 Introduction

Worldwide, the shortage of nursing manpower has become a pressing challenge. In China, the deficit is particularly severe, with only 2.5 nurses per thousand patients, a ratio far below the standard recommended by the World Health Organization (WHO) (Zhang et al., 2023). Clinical nursing education, as a core component of the medical education system, plays a pivotal role in preparing qualified nursing professionals. During this process, nursing interns undergo a substantial transition from theoretical learning to clinical practice, which requires not only mastery of professional knowledge but also the ability to adapt to the complexity and unpredictability of clinical environments. Recent studies, however, have highlighted an issue that warrants close attention: incivility in clinical nursing education (ICNE). ICNE exerts a negative influence on the learning experiences of nursing interns, with learning burnout being particularly concerning (Ahn and Choi, 2019). Learning burnout diminishes the effectiveness of training (Zhao et al., 2022), hinders career development (Shabbir et al., 2020), and poses risks to both physical health (Lee et al., 2014) and psychological well-being (Watson, 2023). Investigating the causes of burnout and identifying strategies to mitigate it are therefore essential for improving the quality of clinical nursing education and fostering the holistic development of nursing interns.

Learning burnout is defined as a state of severe psychological fatigue caused by long-term exposure to academic stress, typically characterized by emotional exhaustion, depersonalization, and a reduced sense of accomplishment (Maslach and Leiter, 2021). This problem is particularly pronounced among nursing interns. Because of the demanding nature of clinical placements, they must rapidly adapt to unfamiliar hospital environments, acquire extensive knowledge and technical skills, and cope with pressures from patients, instructors, and hospital staff. These combined demands substantially increase the risk of burnout. A survey of Chinese nursing interns reported that more than 60% experienced some degree of burnout during training, and nearly 30% noted that burnout severely affected their learning efficiency and enthusiasm. A national study further revealed that 45% of nurses in China experience burnout, with workplace incivility and heavy workload as primary predictors (Zhang et al., 2023). Similarly, Li et al. (2022) found that insufficient organizational support and high patient-to-nurse ratios significantly exacerbate burnout among Chinese nurses. Among nursing interns, psychosocial factors such as low self-efficacy and inadequate mentorship have been identified as important contributors. A nearly study also reported that higher levels of burnout are closely associated with poorer mental health outcomes among nursing interns. Collectively, these findings underscore both the prevalence and severity of learning burnout in this population.

Research has increasingly pointed to ICNE as a critical factor driving learning burnout (Eka and Chambers, 2019). Incivility encompasses behaviors such as verbal insults, discrimination, neglect, indifference, and in extreme cases, physical aggression. Eka and Chambers (2019) reported that more than 40% of nursing students had experienced verbal insults or discrimination during internships, while Zhu et al. (2019) found that nearly 60% of Chinese nursing interns encountered incivility in clinical settings. These findings highlight the widespread nature of the problem. ICNE damages the self-esteem and confidence of nursing interns (Guo et al., 2017), undermines their professional identity and sense of belonging (Yang et al., 2022), and weakens their motivation to learn. Evidence further shows that nursing interns exposed to incivility not only face higher risks of burnout but also tend to experience more severe levels of burnout than their peers (Liu et al., 2023; Zhu et al., 2019). Addressing ICNE is therefore an essential step toward alleviating learning burnout.

Beyond incivility, psychological resources such as career calling and self-efficacy also play a significant role in shaping burnout outcomes. Career calling refers to a transcendent sense of purpose and responsibility toward one’s profession that sustains long-term motivation (Dik and Duffy, 2009). Self-efficacy is defined as confidence in one’s ability to successfully perform tasks and overcome challenges (Bandura, 1977). Both factors are recognized as protective resources against workplace stress. Career calling strengthens resilience by reinforcing professional identity (Dik and Duffy, 2009), while self-efficacy reduces stress by enhancing task mastery (Bandura, 1977). Although their importance is well established, their specific mediating roles in the relationship between ICNE and learning burnout remain insufficiently studied, particularly in hierarchical educational systems where cultural norms may amplify the effects of incivility.

Career calling and self-efficacy function through distinct pathways in mitigating burnout. Career calling nurtures resilience by strengthening professional values and commitment (Dik and Duffy, 2009), while self-efficacy enables individuals to manage stress through confidence in task performance (Bandura, 1977). Recent studies suggest that these two factors may also operate sequentially in educational settings, where career calling enhances self-efficacy, which in turn reduces burnout (Chen et al., 2024; Shang et al., 2022). However, in hierarchical clinical nursing education systems—particularly in non-Western cultural contexts shaped by Confucian values and strong professional ethics—the independent and combined effects of career calling and self-efficacy in buffering ICNE-related burnout remain underexplored.

Grounded in the job demands–resources (JD-R) framework (Bakker and Demerouti, 2017) and supported by emerging evidence on burnout mitigation pathways (Chen et al., 2024; Shang et al., 2022), this study hypothesizes three relationships: (1) ICNE is positively associated with learning burnout. (2) Career calling mediates the relationship between ICNE and learning burnout. (3) Self-efficacy mediates this relationship.

Most prior studies have predominantly focused on the direct effects of ICNE on learning burnout (Zhang et al., 2023). However, the mediating influence of career calling and self-efficacy remains poorly understood, particularly in cultural settings where Confucian values may uniquely shape responses to incivility.

This study makes two contributions: (1) It identifies the dual mediating pathways of career calling and self-efficacy in the relationship between ICNE and learning burnout, and (2) it provides practical implications for educators by suggesting culturally tailored interventions, such as structured mentorship and resilience-building programs, that may help mitigate burnout. In doing so, the study extends the JD-R model by integrating culturally specific psychological resources into its explanatory framework.

## 2 Research methods

This study aimed to comprehensively examine the impact of ICNE on learning burnout among Chinese nursing interns, with particular emphasis on the mediating effects of career calling and self-efficacy.

### 2.1 Research participants

A total of 300 nursing interns were invited to participate in the survey, of whom 268 responded (response rate: 89.3%). After excluding 18 invalid questionnaires, 250 valid responses were retained for analysis. Hospital records confirmed that non-respondents (*n* = 32) did not differ significantly from respondents in terms of age, gender, or education level (*p* > 0.05). The minimum sample size was estimated using two approaches. First, based on the widely accepted rule of 5–10 participants per scale item in structural equation modeling (Comrey and Lee, 1992), the 12-item ICNE scale required a minimum of 60–120 participants. Second, an *a priori* power analysis using G*Power 3.1 with *α* = 0.05, medium effect size (Cohen’s f^2^ = 0.15), and power = 0.95 indicated a required sample size of 200 (Babenko-Mould and Laschinger, 2014). Considering a potential 20% attrition rate due to invalid or incomplete responses, the target sample size was set at 240. A *post hoc* power analysis confirmed that the final sample (*N* = 250) achieved 97% statistical power, exceeding the recommended threshold.

Inclusion criteria were nursing interns who (1) had completed at least 7 months of clinical internship; (2) could independently understand and complete the questionnaire, and (3) voluntarily signed the informed consent form. The exclusion criteria were those (1) on sick or personal leave during the survey period; (2) with severe physical or mental health conditions affecting clinical training; and (3) unwilling or unable to complete the questionnaire.

The study was conducted at a tertiary teaching hospital in Hunan Province, China. The institution provides a clinical training environment comparable to other urban hospitals nationwide, although regional cultural and socioeconomic differences may limit the generalizability of findings to rural or non-urban contexts. Ultimately, 268 questionnaires were collected, and after excluding those with more than 20% missing responses or inconsistencies in reverse-scored items, 250 valid responses were analyzed.

### 2.2 Research instruments

#### 2.2.1 Demographic data questionnaire

A demographic questionnaire was designed based on prior literature, including items on age, gender, ethnicity, only-child status, education level, reasons for choosing nursing, and place of origin.

#### 2.2.2 ICNE scale

The ICNE scale, developed by Anthony et al. (2014), contains 12 items across two dimensions: disdainful behavior (7 items) and exclusive behavior (5 items). Responses are rated on a 5-point Likert scale (0 = never occurred to 4 = frequently occurred), yielding total scores from 0 to 48. Higher scores reflect greater severity of incivility. Example items include “Embarrass you in front of others” (disdainful behavior) and “Not letting you know what you should have known about your patients” (exclusive behavior). The Chinese version demonstrated excellent reliability, with Cronbach’s *α* = 0.96 for the total scale and 0.95 and 0.93 for the two subscales.

#### 2.2.3 Learning Burnout Scale

The Learning Burnout Scale measures three dimensions: emotional exhaustion (8 items), inappropriate behavior (6 items), and low sense of achievement (6 items). Responses are rated on a 5-point Likert scale (1 = completely disagree to 5 = completely agree), with 8 items reverse-scored. Example items include “I find what I have learned useless” (low sense of achievement) and “I feel very tired thinking about facing a day of study” (emotional exhaustion). Total scores range from 0 to 80 and are categorized into five burnout severity levels. The scale showed high reliability, with Cronbach’s *α* = 0.92 overall and 0.85, 0.79, and 0.78 for the three subscales, respectively.

#### 2.2.4 Career Calling Scale

The Career Calling Scale (Dobrow and Tosti-Kharas, 2011) is a unidimensional 12-item measure rated on a 5-point Likert scale (1 = strongly disagree to 5 = strongly agree). Representative items include “I’m passionate about nursing” and “Nursing gives me great personal satisfaction.” The original validation study across four disciplines (music, business, art, management) reported Cronbach’s *α* between 0.88 and 0.94. The Chinese adaptation validated in nursing populations (Pei et al., 2015) confirmed strong reliability and validity.

#### 2.2.5 General Self-Efficacy Scale

The General Self-Efficacy Scale includes 10 items rated on a 4-point Likert scale (1 = completely incorrect to 4 = completely correct). Example items include “I have the ability to correctly perform basic nursing procedures” and “I am confident in solving problems independently during my nursing internship.” The Chinese adaptation has been widely applied in nursing research, showing high internal consistency (α > 0.85) and good test–retest reliability over 2 weeks (*r* = 0.78).

### 2.3 Data collection

All responses were anonymized to ensure confidentiality. A pilot test with 10 nursing interns confirmed questionnaire clarity and reliability using a paper-based format. Formal data collection was conducted online. A QR code linking to the digital questionnaire was distributed via the WeChat management group. Participants scanned the code, provided informed consent, and submitted responses within a 30-min window. The average completion time was 15 ± 3.2 min. The system restricted submissions to one per participant, and researchers monitored the process to prevent collaboration. To minimize common method bias, measures included anonymous participations and reverse-scored items such as “I am full of energy when I study” from the Learning Burnout Scale. Questionnaires with more than 20% missing responses or logically inconsistent answers to reverse-scored items were excluded. Missing data, which represented less than 2% per variable, were handled by listwise deletion.

### 2.4 Data analysis

Data analysis was conducted using SPSS 27.0 and AMOS 26.0. Descriptive statistics were used to summarize demographic characteristics and variable distributions. Harman’s single-factor test indicated no significant common-method bias (first-factor variance = 38.7%, below the 50% threshold). Pearson correlation analysis was performed to examine relationships among variables. Regression analysis assessed the effect of ICNE on learning burnout while controlling for covariates. Structural equation modeling (SEM) with a bias-corrected bootstrap method was employed to evaluate both direct and mediating effects. Model fit indices indicated good fit: χ^2^/df = 1.696, IFI = 0.996, TLI = 0.992, CFI = 0.996, and RMSEA = 0.053.

## 3 Results

### 3.1 Demographic description

The participants in this study were aged 15–28 years, with a mean age of 20.25 ± 2.62 years. Females constituted 88.8% of the sample. Educational attainment was distributed as follows: junior college (associate degree) or below, 50.4%; bachelor’s degree, 35.2%; and master’s degree or above, 14.4%. Most participants were of Han ethnicity (90.8%), while 9.2% belonged to ethnic minority groups. Regarding family structure, 62.4% came from multi-child families. The motivations for choosing nursing varied, with 30% citing personal interest, 25% family influence, and 45% career prospects. The sample was almost evenly divided between urban (49.2%) and rural (50.8%) backgrounds. With respect to career intentions, 79.6% planned to pursue nursing or related fields, while 20.4% intended to pursue non-nursing careers. Nearly half of the participants (48%) had relatives in the nursing profession, and 70.8% were first-time interns. Detailed demographic data are presented in Table 1.

**Table 1:** Demographic characteristics of nursing interns and analysis of demographic differences among variables (*N* = 250).

Demographics	*N*	%	ICNE	CC	SE	LB
Gender						
Male	28	11.2	17.36 ± 6.90	31.14 ± 3.62	22.71 ± 2.27	56.29 ± 7.54
Female	222	88.8	16.91 ± 7.18	31.42 ± 3.56	22.86 ± 2.13	55.71 ± 6.97
t			0.312	−0.329	−0.329	0.407
Age (y)						
<18	46	18.4	21.17 ± 7.78	29.74 ± 3.86	21.39 ± 2.39	60.00 ± 8.63
≥18	204	81.6	16.01 ± 6.64	31.76 ± 3.39	23.17 ± 1.94	54.82 ± 6.24
t			4.612	−3.562*	−5.36*	4.707**
Education						
Junior college or below	126	50.4	18.41 ± 6.72	30.67 ± 3.41	22.46 ± 2.07	57.29 ± 6.77
Bachelor’s degree	88	35.2	16.99 ± 6.46	31.33 ± 3.14	22.74 ± 1.88	55.73 ± 6.46
Master’s degree or above	36	14.4	11.81 ± 7.82	34.08 ± 3.86	24.42 ± 2.30	50.61 ± 6.90
F			13.188**	14.256**	13.004**	13.986**
Ethnicity						
Han	227	90.8	17.28 ± 7.01	31.39 ± 3.55	22.82 ± 2.14	55.98 ± 7.11
Ethnic minority	23	9.2	13.83 ± 7.74	31.43 ± 3.79	23.04 ± 2.18	53.78 ± 5.80
t			2.229	−0.060	−0.478	1.432
Only Child						
Yes	94	37.6	21.52 ± 6.26	29.45 ± 3.17	21.85 ± 2.20	60.09 ± 7.14
No	156	62.4	14.21 ± 6.16	32.56 ± 3.27	23.44 ± 1.87	53.18 ± 5.52
t			9.028	−7.383	−6.068	8.560*
Reasons for Choosing Nursing Major						
Voluntary	90	36	15.00 ± 7.39	32.94 ± 3.93	23.54 ± 2.19	53.61 ± 6.90
Family’s Wish	74	29.6	17.00 ± 5.82	31.05 ± 2.77	22.77 ± 1.88	55.55 ± 5.64
Adjustment	72	28.8	18.93 ± 7.44	29.97 ± 3.09	22.15 ± 2.11	58.25 ± 7.43
Other	14	5.6	19.21 ± 7.39	30.05 ± 3.59	22.21 ± 2.01	58.14 ± 8.03
F			4.771*	11.249**	6.568**	6.842**
Family Address						
Urban	123	49.2	17.28 ± 7.47	31.12 ± 3.57	22.62 ± 2.16	56.21 ± 7.43
Rural (Township)	127	50.8	16.65 ± 6.81	31.65 ± 3.56	23.06 ± 2.11	55.35 ± 6.60
t			0.69	−1.180	−1.621	0.965
Employment Intentions						
Nursing and Related	199	79.6	16.31 ± 6.74	31.72 ± 3.46	23.13 ± 2.02	55.01 ± 6.40
Non-Nursing and Related	51	20.4	19.51 ± 8.09	30.12 ± 3.72	21.73 ± 2.25	58.76 ± 8.47
t			−2.904	2.904	4.315	−3.484 *
Family Member in Nursing Profession						
Yes	120	48	13.68 ± 6.24	33.29 ± 3.13	23.75 ± 1.93	52.74 ± 5.44
No	130	52	19.99 ± 6.55	29.64 ± 3.00	22.00 ± 1.98	58.58 ± 7.16
t			−7.789	9.413	7.069	−7.208*
Previous Internship Experience						
Yes	73	29.2	16.49 ± 8.06	31.84 ± 3.95	23.08 ± 2.34	55.03 ± 7.60
No	177	70.8	17.15 ± 6.73	31.21 ± 3.39	22.75 ± 2.05	56.08 ± 6.76
t			−0.664*	1.265*	1.085	−1.083

*p<0.05, **p<0.01, ICNE, incivility in clinical nursing education; CC, career calling; SE, self-efficacy; LB, learning burnout.

### 3.2 Descriptive statistics of variables

The primary variables analyzed in this study were ICNE, career calling, general self-efficacy, and learning burnout (Table 2). The mean ICNE score was 16.96 ± 7.13, suggesting that nursing interns experienced mild to moderate levels of incivility in clinical education, with considerable variability. ICNE scores differed significantly by education level, reasons for choosing nursing, and internship experience (*p* < 0.05). The mean learning burnout score was 55.78 ± 7.02, indicating a moderately high level of burnout, with some participants experiencing severe burnout. Learning burnout scores varied significantly across age, education level, only-child status, reasons for choosing nursing, career intentions, and whether family members were in the nursing profession (*p* < 0.05). The mean career calling score was 31.39 ± 3.56, reflecting a moderate level of career calling among most interns. Significant differences were observed across age, education level, reasons for choosing nursing, and internship experience (*p* < 0.05). The mean self-efficacy score was 22.84 ± 2.14, indicating a moderate level of self-efficacy with relatively low variability. Self-efficacy scores differed significantly across age, education level, and reasons for choosing nursing (*p* < 0.05). Results are summarized in Table 2.

**Table 2:** Descriptive analysis of variable scores.

Variable	Number of items	Scale range	Observed range	Mean ± SD	Interpretation
ICNE	12	0–48	0–34	16.96 ± 7.13	Mild to moderate incivility
CC	12	12–60	24–39	31.39 ± 3.56	Moderate career calling
SE	10	10–40	17–29	22.84 ± 2.14	Moderate self-efficacy
LB	20	20–100	40–74	55.78 ± 7.02	Moderately high burnout

*p<0.05, **p<0.01, ICNE, incivility in clinical nursing education; CC, career calling; SE, self-efficacy; LB, learning burnout.

### 3.3 Correlations between variables

As shown in Table 3, all four variables were significantly correlated. ICNE was negatively correlated with both career calling and self-efficacy, and positively correlated with learning burnout. Learning burnout was negatively correlated with career calling and self-efficacy, whereas career calling was positively correlated with self-efficacy.

**Table 3:** Pearson correlation analysis results of incivility in clinical nursing education, career calling, learning burnout, and self-efficacy(r).

Variables	ICNE	CC	SE	LB
ICNE	1			
CC	−0.847**	1		
SE	−0.749**	0.740**	1	
LB	0.878**	−0.829**	−0.794**	1

ICNE, incivility in clinical nursing education; CC, career calling; SE, self-efficacy; LB, learning burnout. **indicates that the correlation coefficient is statistically significant at the 0.01 level (2-tailed) in the Pearson correlation analysis, i.e., *p* < 0.01.

### 3.4 Regression analysis

Regression analysis results are presented in Table 4. ICNE and selected demographic factors significantly influenced learning burnout. The overall regression model was significant (*F* = 88.02, *p* < 0.001), explaining 78.6% of the variance in learning burnout. Significant demographic covariates included age, education level, and only-child status, with only-child status remaining significant after adjustment (*β* = 0.102, *p* < 0.05). ICNE remained a strong predictor of learning burnout, with coefficients stable before and after adjustment (*β* = 0.784 vs. *β* = 0.772). Specifically, ICNE was positively associated with learning burnout (*t* = 19.876, *p* < 0.001). These findings indicate that incivility in clinical nursing education is closely linked to higher levels of learning burnout among interns.

**Table 4:** Path relationship test.

Path	Estimate	S. E.	C. R.	*p*
Incivility → Self-Efficacy	−0.755	0.032	−17.449	<0.001
Incivility → Career Calling	−0.836	0.045	−22.545	<0.001
Self-Efficacy → Learning Burnout	−0.268	0.052	−6.043	<0.001
Incivility → Learning Burnout	0.512	0.049	8.896	<0.001
Incivility → Learning Burnout	−0.322	0.037	−6.011	<0.001

C. R. = critical ratio; Lower/Upper = 95% Confidence Interval bounds. Adjusted for age, education level, and only-child status.

### 3.5 SEM

**Figure 1:**
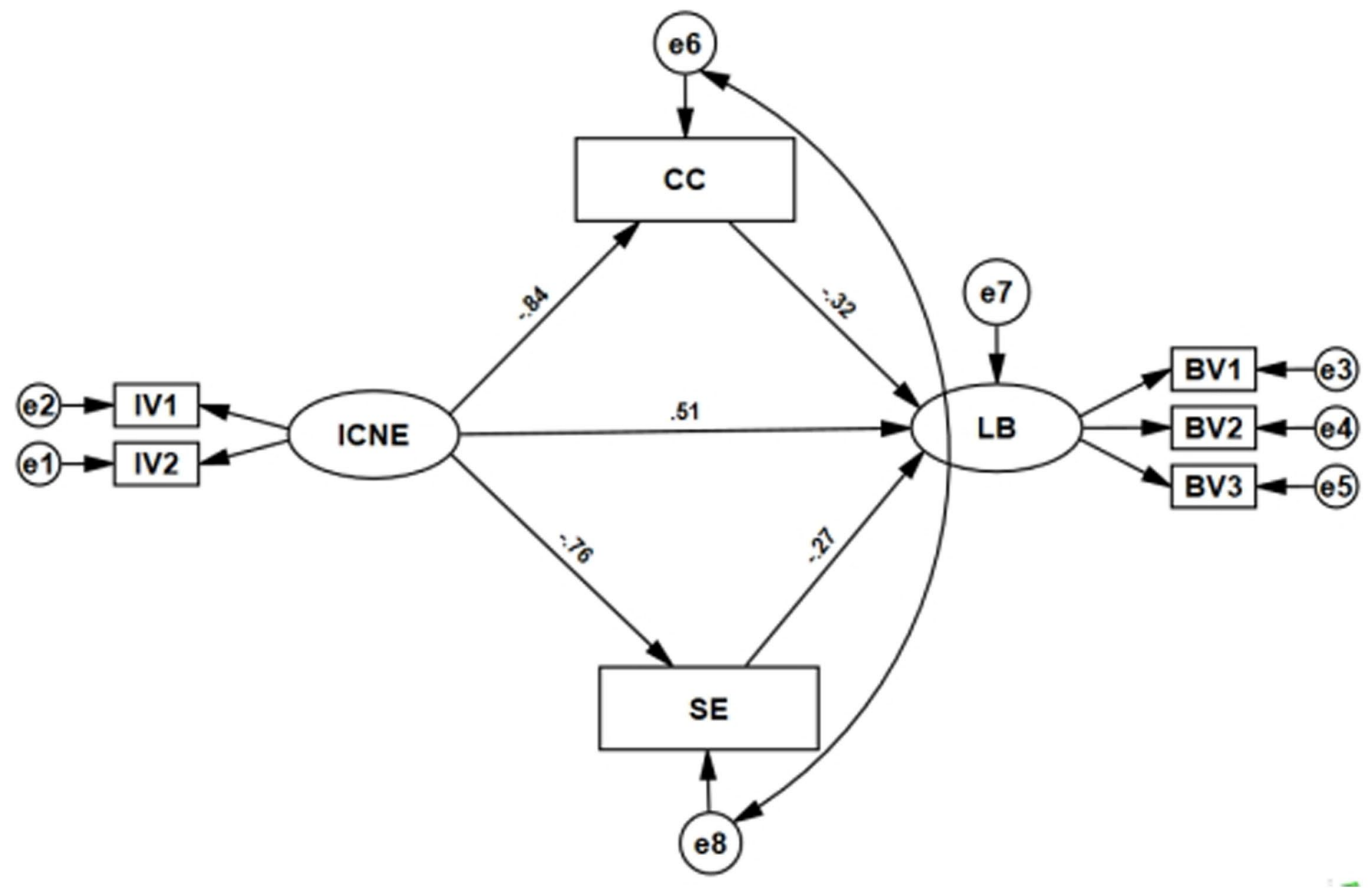
Structural equation model of the effects of incivility on learning burnout with mediating roles of career calling and self-efficacy.

AMOS was used to test the mediating effects of career calling and self-efficacy. After a single model adjustment, residual 6 was correlated with residual 8 (Figure 1), and all fit indices met acceptable standards (Table 5). Path analysis (Table 4) showed that all five model paths were statistically significant. The results demonstrated that higher levels of perceived incivility were associated with lower self-efficacy and career calling, and with higher learning burnout. Bootstrap mediation analysis confirmed partial mediation (Table 6). The indirect effect of career calling (indirect effect 1) was significant, accounting for 27.3% of the total effect. The indirect effect of self-efficacy (indirect effect 2) was also significant, accounting for 20.6% of the total effect. Together, the indirect effects explained 47.9% of the variance, while the direct effect of incivility on learning burnout accounted for 52.1%. There was no statistically significant difference between the contributions of the two mediators (*p* = 0.387).

**Table 5:** Model fit test.

Indicator	Reference standard	Actual value
CMIN/DF	1–3 is excellent, 3–5 is acceptable	1.696
RMSEA	<0.05 is excellent, <0.08 is acceptable	0.053
IFI	>0.9 is excellent, >0.8 is acceptable	0.996
TLI	>0.9 is excellent, >0.8 is acceptable	0.992
CFI	>0.9 is excellent, >0.8 is acceptable	0.996

**Table 6:** The bootstrap intermediary efficacy test results.

Parameter	Estimate	Lower	Upper	*p*	Effect ratio
Indirect Effect 1	0.227	0.132	0.321	0.001	27.3%
Indirect Effect 2	0.171	0.106	0.238	0.001	20.6%
Total Indirect Effect	0.399	0.286	0.502	0.001	47.9%
Direct Effect	0.433	0.311	0.572	0.001	52.1%
Total Effect	0.832	0.73	0.928	0.001	100%
Total Effect	0.056	−0.076	0.172	0.387	

## 4 Discussion

This study examined the incivility experienced by Chinese nursing interns during clinical education, its impact on learning burnout, and the mediating roles of career calling and self-efficacy. The results demonstrated a significant positive association between incivility and learning burnout, with both career calling and self-efficacy serving as important mediators. These findings provide valuable evidence for understanding the effects of incivility on the psychological well-being and professional development of nursing interns.

### 4.1 Discussion of main variable results

The mean incivility score reported by nursing interns was 16.96 ± 7.13, reflecting a mild to moderate level of perceived incivility with substantial variability. This finding is consistent with Ahn and Choi (2019), who similarly documented the prevalence of incivility in nursing education. Incivility has been reported across cultural contexts (Kim et al., 2024) and is known to adversely affect students’ learning experiences and psychological health, underscoring the urgency of addressing this issue. In the Chinese context, incivility may be linked not only to traditional hierarchical teaching models but also to stress, heavy workloads, and poor communication in clinical education. The present study also identified differences in incivility scores across demographic groups, particularly by education level and reasons for choosing nursing. Interns with higher education levels may be more aware of dignity and rights, making them more sensitive to incivility, whereas those who entered nursing under family pressure or by default may have weaker professional identities and thus perceive greater incivility. These findings highlight the importance of tailoring support to the individual backgrounds and developmental needs of interns, offering personalized support and assistance.

The total learning burnout score was 55.78 ± 7.02, indicating a moderately high level overall, with some interns experiencing markedly severe burnout. This result supports previous findings (Ghods et al., 2023; Wu et al., 2021) showing that burnout is a common issue among nursing interns, often linked to learning pressure, demanding workloads, and stressful clinical environments. Some interns may feel overwhelmed by academic and clinical demands, leading to disengagement and fatigue. These results emphasize the need for nursing educators to monitor interns’ learning status, implement strategies to reduce pressure, and enhance engagement in clinical education.

Interns generally reported moderate levels of career calling and self-efficacy, consistent with findings from Jiang et al. (2023) and Bulfone et al. (2022). Both constructs were shown to buffer burnout (Chen and Wang, 2019). In the Chinese cultural context, career calling is reinforced by Confucian values that emphasize professional ethics and social responsibility, thereby strengthening professional identity. Meanwhile, self-efficacy enhances confidence in clinical competence and resilience in stressful environments. Thus, clinical nursing education should incorporate individualized career guidance and structured support to cultivate career calling and self-efficacy.

### 4.2 Discussion on relationships between variables

The results revealed a significant negative correlation between incivility and both career calling and self-efficacy, and a significant positive correlation between incivility and learning burnout. These findings are consistent with Jiang et al. (2023) and Pitacho and Cordeiro (2023), who reported that incivility undermines career calling and self-efficacy, thereby exacerbating burnout. Previous research has also shown that interventions promoting self-efficacy and professional identity buffer the effects of workplace incivility (Lee et al., 2019). However, studies such as Chen et al. (2019) suggest that in highly authoritarian educational contexts, these interventions may be less effective, emphasizing the need for context-specific approaches. Additionally, this study found a significant negative correlation between learning burnout and both career calling and self-efficacy and a significant positive correlation between career calling and self-efficacy. This finding indicates that interns with stronger professional confidence and calling are more motivated and self-disciplined in their professional studies.

Regression analysis further demonstrated that incivility positively predicted learning burnout (*β* = 0.784, *p* < 0.001), accounting for 78.6% of the variance. This aligns with Babenko-Mould and Laschinger (2014), who also identified incivility as a strong predictor of burnout. Additionally, being an only child significantly influenced burnout levels, possibly due to the absence of sibling support and heightened family expectations under China’s one-child policy (Watson, 2023). Interns from single-child families may rely more heavily on individual coping mechanisms, making them more vulnerable to stress and burnout. These findings highlight the importance of providing additional psychological and educational support for this demographic group.

### 4.3 Discussion of mediation effect analysis

Structural equation modeling revealed that career calling and self-efficacy partially mediated the relationship between incivility and learning burnout. This finding offers a new perspective for understanding the mechanism by which incivility in nursing education affects learning burnout. Specifically, incivility directly contributed to 52.1% of the total effect on burnout, with indirect effects mediated by career calling (27.3%) and self-efficacy (20.6%). These findings are consistent with studies emphasizing the buffering role of intrinsic motivation in adverse environments (Watson, 2023; Wu et al., 2021). Specifically, the mediating effects of career calling and self-efficacy highlight the crucial role of intrinsic motivation and confidence when interns face incivility. Career calling allows interns to sustain motivation and a sense of professional responsibility toward their profession when facing challenges, while self-efficacy enables them to cope more effectively with stressors. Together, these mediators highlight the protective role of internal resources in mitigating negative educational experiences. Therefore, enhancing career calling and self-efficacy through career values education, clinical practice opportunities, timely feedback and support, and improved teamwork and communication should be prioritized in nursing education to strengthen interns’ resilience and career development.

### 4.4 Comparative analysis with other studies

Consistent with global literature, this study confirms that incivility significantly predicts learning burnout (*β* = 0.784, *p* < 0.001), echoing findings from Western contexts (Babenko-Mould and Laschinger, 2014). However, the present study extends prior knowledge by quantifying the mediating effects of career calling and self-efficacy, which together accounted for 47.9% of the incivility–burnout relationship. Although Lopes and Nihei (2020) emphasized the buffering role of self-efficacy, they did not specify its proportion in the overall mechanism.

Cultural context also plays a critical role. Whereas Western studies have highlighted external support systems (Kim et al., 2024), Chinese interns appear to rely more on internal motivators such as career calling, reflecting Confucian values that prioritize duty and perseverance. This cultural influence may explain the comparatively larger mediating role of career calling (27.3%) observed in our model compared with reports from more individualistic cultures (Kim et al., 2021).

The finding that career calling and self-efficacy independently mediated the incivility–burnout relationship aligns with Shang et al. (2022), who observed dual pathways in preservice teachers. However, unlike Shang et al., who reported sequential mediation (career calling leading to self-efficacy), our model supports parallel effects, suggesting that in nursing interns, calling and self-efficacy operate independently. This difference may be linked to Confucian collectivism, which emphasizes professional responsibility as a standalone value.

These findings underscore the need for cross-cultural replications. For example, whereas our results suggest that Chinese interns rely primarily on internal regulation, studies from other collectivist contexts, such as South Korea, highlight the importance of peer networks (Riley et al., 2021). Future research should investigate how cultural norms and educational policies interact to shape responses to incivility across different settings.

### 4.5 Limitations and future research directions

This study has several limitations. First, the sample comprised nursing interns from a single region in China, limiting the generalizability of the findings to broader national or cross-cultural contexts. Although the hospital’s clinical training environment was consistent with that of urban teaching hospitals, regional and cultural differences may restrict applicability to rural or less resourced settings. Second, the cross-sectional design prevents causal inference, and reverse causality (e.g., burnout influencing perceptions of incivility) cannot be excluded. Third, the exclusive use of self-reported data may have introduced response bias. Fourth, confirmatory factor analysis (CFA) was not performed to validate the measurement structures, thereby limiting verification of convergent and discriminant validity. The decision not to conduct CFA was methodologically justified by three considerations: (1) sample size limitations, as robust CFA for multidimensional scales requires larger samples than mediation models. For instance, Wolf et al. (2013) recommend at least 10 participants per item (e.g., 120 for the 12-item ICNE), whereas our sample (*N* = 250) was allocated to ensure adequate power for path analysis rather than psychometric testing (MacCallum et al., 1999); (2) established alternatives, given that validated scales with high internal consistency (*α* > 0.85) and theoretically consistent correlation patterns (e.g., ICNE–career calling *r* = −0.847) provide sufficient evidence for model testing without CFA (Podsakoff et al., 2012); and (3) cross-cultural precedent, since the Chinese adaptations of these measures have shown strong metric invariance in prior studies (CFI = 0.98; Lian et al., 2005: RMSEA = 0.04), reducing the immediate need for revalidation (Cheung and Rensvold, 2002). Future research should therefore extend sampling to diverse geographic and healthcare settings, employ longitudinal or experimental designs to clarify causality, and integrate multi-source or objective data to reduce bias. Additionally, CFA should be incorporated in future studies to strengthen psychometric evaluations, particularly when adapting measures across culturally distinct populations.

### 4.6 Theoretical implications

This study advances theoretical understanding of the mechanisms linking incivility with learning burnout in nursing education. By identifying career calling and self-efficacy as significant mediators, accounting for 27.3 and 20.6% of the indirect effects respectively, our findings validate and extend the JD-R model (Bakker and Demerouti, 2017), emphasizing the role of intrinsic motivators in buffering workplace stressors. Furthermore, the integration of career calling within the Chinese cultural context, shaped by Confucian values that prioritize professional ethics and social responsibility, offers a culturally nuanced perspective on vocational identity (Dik and Duffy, 2009). This contribution addresses a gap in the literature, which has primarily focused on Western, individualistic frameworks.

### 4.7 Practical implications

The findings have important implications for clinical nursing education in China. First, enhancing career calling—identified as the stronger mediator (27.3%)—requires embedding Confucian virtues such as ren (benevolence) and professional ethics into mentorship programs. Clinical instructors should model compassionate care and employ narrative pedagogy to illustrate how preserving patient dignity can overcome workplace adversity. Second, interventions to strengthen self-efficacy should be context-specific. Structured simulation training should replicate high-stress clinical situations, particularly those in emergency department rotations where incivility was most frequently reported. Third, institutional responses should move beyond punitive measures by embedding principles of mutual respect into teacher certification standards and implementing anonymous, real-time feedback systems to systematically reduce incivility. Finally, targeted support for single-child interns—who reported 12.3% higher levels of burnout—could be developed through family-school collaboration models rooted in Chinese collectivist traditions, encouraging parental engagement in resilience-building efforts.

In summary, this study demonstrates that incivility significantly exacerbates learning burnout among Chinese nursing interns and identifies career calling and self-efficacy as key protective mechanisms. These findings contribute to both theoretical frameworks and practical strategies for fostering resilience in clinical education.

## 5 Conclusion

This study examined the effects of ICNE on learning burnout among Chinese nursing interns and evaluated the mediating roles of career calling and self-efficacy. Results indicate that incivility significantly increases burnout levels, while both career calling and self-efficacy serve as critical mediators in this process. These findings provide important insights into the impact of incivility on the psychological well-being and career development of nursing interns.

First, the study confirmed that incivility is prevalent in clinical nursing education and has a detrimental effect on interns’ learning experiences. Interns exposed to incivility exhibited higher levels of burnout, consistent with previous research, underscoring the importance of improving teacher–student relationships and cultivating a supportive educational environment.

Second, the mediating roles of career calling and self-efficacy were demonstrated, with higher levels of each associated with reduced burnout in the face of incivility. This suggests that fostering career calling and strengthening self-efficacy can help mitigate the negative impact of incivility. Therefore, nursing education should prioritize the cultivation of these protective factors to support interns in developing a strong professional identity and confidence in their abilities.

In conclusion, this study highlights the significant influence of ICNE on interns’ learning burnout and proposes strategies to address this issue through the mediating roles of career calling and self-efficacy. Future research should investigate additional mediating factors, such as social support and coping strategies, to provide a more comprehensive understanding of how incivility affects learning burnout. At the institutional level, efforts should focus on promoting the psychological well-being of interns, implementing effective measures to reduce incivility, and enhancing the overall quality of nursing education to ensure the development of competent and resilient nursing professionals.

## Data Availability

The original contributions presented in the study are included in the article/supplementary material, further inquiries can be directed to the corresponding author.
